# Hound: a novel tool for automated mapping of genotype to phenotype in bacterial genomes assembled *de novo*

**DOI:** 10.1093/bib/bbae057

**Published:** 2024-02-21

**Authors:** Carlos Reding, Naphat Satapoomin, Matthew B Avison

**Affiliations:** University of Bristol School of Cellular and Molecular Medicine, University Walk, Bristol, BS8 1TD Bristol, UK; University of Bristol School of Cellular and Molecular Medicine, University Walk, Bristol, BS8 1TD Bristol, UK; University of Bristol School of Cellular and Molecular Medicine, University Walk, Bristol, BS8 1TD Bristol, UK

**Keywords:** genomic surveillance, antibiotic resistance, reference-free

## Abstract

Increasing evidence suggests that microbial species have a strong within species genetic heterogeneity. This can be problematic for the analysis of prokaryote genomes, which commonly relies on a reference genome to guide the assembly process. Differences between reference and sample genomes will therefore introduce errors in final assembly, jeopardizing the detection from structural variations to point mutations—critical for genomic surveillance of antibiotic resistance. Here we present Hound, a pipeline that integrates publicly available tools to assemble prokaryote genomes *de novo*, detect user-given genes by similarity to report mutations found in the coding sequence, promoter, as well as relative gene copy number within the assembly. Importantly, Hound can use the query sequence as a guide to merge contigs, and reconstruct genes that were fragmented by the assembler. To showcase Hound, we screened through 5032 bacterial whole-genome sequences isolated from farmed animals and human infections, using the amino acid sequence encoded by *bla_TEM-1_*, to detect and predict resistance to amoxicillin/clavulanate which is driven by over-expression of this gene. We believe this tool can facilitate the analysis of prokaryote species that currently lack a reference genome, and can be scaled either up to build automated systems for genomic surveillance or down to integrate into antibiotic susceptibility point-of-care diagnostics.

## Graphical Abstract

**Graphical Abstract:**
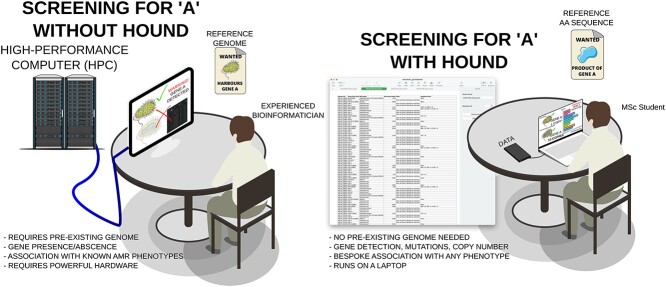

## INTRODUCTION

The advent of affordable genome sequencing has exposed the wide genetic heterogeneity that exists within bacterial species [1]. With genome sizes that range between 2.69 and 2.92 Mb in *Staphylococcus aureus*, or between 4.66 and 5.30 Mb for *Escherichia coli*, it is not surprising that some begin to question the notion of *species* [2, 3] or even *clone* [4] in prokaryotes. This heterogeneity led to the concept of *pan-genomes* [5], but it also exposes another, more technical problem: How to study the genomes of prokaryotes without masking this genetic diversity?

Raw sequencing data are typically mapped onto a high-quality reference—whose sequence is known and resolved (i.e. circularized) [6, 7]—or databases containing them [8], to study the genetics of organisms from viruses [9] to vertebrates [10] or plants [11]. The use of reference-mapped assemblies is used in comparative genomics [12], clinical microbiology [13], public health [14, 15], and even to inform policy through the detection of specific mutations or phylogenetic analyses [16, 17]. Now, given the further reduction in sequencing costs, reference-mapped assemblies are increasingly used to predict antibiotic susceptibility in bacteria from clinical samples [18, 19]. This is driven by the ability of genomics to screen resistance to multiple antibiotics simultaneously, more than is possible with current phenotypic antibiotic sensitivity tests, improving antibiotic stewardship and patient care. But using genomics data for this can be problematic, given the limitations of these type of assemblies to detect antibiotic-resistance (ABR) genes. On one hand, reads that cannot be mapped onto the reference genome, say, because they are plasmid-borne and not part of the chromosome, are excluded from the assembly. And this loss of data hinders the detection of ABR genes [19, 20]. On the other hand, the availability of reference genomes is skewed toward the most common pathogens [13, 16], further limiting the study of rarer pathogens [21]. Consequently, the scope of tools like ResFinder [22], STARR [23], ARG-ANNOT [24], RAST [25] or ABRIcate (https://github.com/tseemann/abricate) can be limited to predict antibiotic susceptibility. Particularly, because they rely on reference genomes to report the presence—or not—of ABR genes along with mutations in the coding sequence *known* to be associated with specific resistant phenotypes. As we show below, mapping sequencing data onto a reference genome can artificially modify the assembly [20]. This approach is not only limited for the study of other pathogens or the finding of novel, undocumented mutations associated with important phenotypes like antibiotic resistance; but of species that may have other biological or ecological importance where reference genomes and tools are scarce [26].

Here we sought to build a pipeline to analyze bacterial genomes assembled *de novo*, without using a reference to guide the assembly process. *De novo* assemblies lack most of the limitations mentioned above, but can also introduce others. Particularly, the fragmentation of genes—whose sequences are split across multiple contigs by the assembler [19]. Hound implements an algorithm to re-purpose a query sequence as a local reference to detect and merge the relevant contigs, so that its sequence can be reconstructed unambiguously. Another issue we sought to address is that antibiotic resistance is not only caused by the presence of specific genes. The over-expression of antibiotic resistance genes, whether through specific mutations in the promoter or increase in relative gene copy number, can dramatically alter the antibiotic resistance phenotype present. For example, amoxicillin-resistant *E. coli* are most commonly resistant due to the production the TEM $\beta -$lactamase enzyme, encoded by the mobile gene *bla_TEM-1_* [27]. Amoxicillin given in combination with clavulanate will kill amoxicillin-resistant *E. coli* because clavulanate inhibits TEM-1—as well as other related enzymes—explaining why this combination has been widely used in human [28, 29] and veterinary medicine [30]. However, *E. coli* can become resistant to amoxicillin-clavulanate by over-producing TEM-1 due to promoter mutations [28] or increased gene copy number [28, 31]. Therefore, we built into Hound the capability to retrieve sequences beyond a gene’s coding sequence and include the promoter, as well as the relative gene copy number, to allow the detection of such variants with our pipeline.

## RESULTS

### Pipeline overview

**Figure 1:**
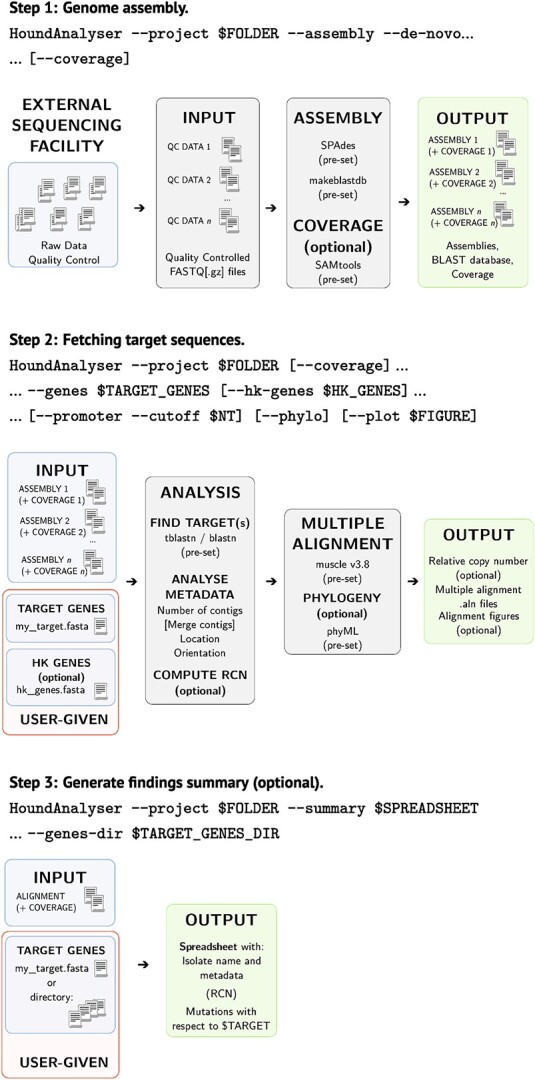
Description of the Hound pipeline for the analysis of bacterial genomes assembled de novo. While Hound can be run in a single step, the user is given three steps of granularity. 1) The first step is to assemble the quality-filtered FASTQ files and depth of coverage data generated. During this step, each assembly is converted into a BLAST database to facilitate downstream analyses. Note that once assembled, there is no need to repeat the step—whence the choice of granularity. 2) In the second step, Hound will search by similarity any user-given target(s) in the assemblies generated in 1). The target sequence(s) must be in FASTA format and preferably be the amino acid sequence to avoid variations introduced by synonymous mutations. Files with multiple entries are supported. To compute the relative gene copy number (RCN), Hound will use a number of house-keeping genes (HK) to compute a baseline depth of coverage. We used four but there is no limit in number of house-keeping genes. If the identity of the sequence found in the assembly with respect to the target, translated as necessary, is above 90%, and the sequence is fragmented, Hound will use the user-given sequence as a guide to sort, de-duplicate, and merge the relevant contigs so the resulting translation is the query sequence used. All sequences are then aligned to facilitate the screening of mutations, insertions or deletions with respect to the target sequence. 3) The last step is optional, and invokes Hound to summarize all the findings from 2) into one spreadsheet for record-keeping.

Hound integrates tools widely used to assemble Nanopore and Illumina reads *de novo*, and screen the resulting assemblies for user-given query sequences, into a single tool. Hound supports nucleotide and amino acid sequences, but we suggest the query to be an amino acid sequence where possible to avoid variations introduced by synonymous mutations. Our pipeline is modular as Figure 1 illustrates to allow performing only a subset of the tasks, and relies on SPAdes [32] as its backend assembler due to its combination of speed, accuracy and support for sequencing data from multiple platforms. Once assembled, Hound can optionally map the raw reads onto the assembly using Burrows-Wheeler aligner [33] to compute the coverage depth with SAMtools [34]. Following the assembly step Hound will search for the query sequences in the assembly by similarity, using the BLAST [35] algorithm, before undergoing downstream processing. At this point, Hound will integrate the data to estimate the relative gene copy number, retrieve sequences upstream of the coding sequence in the assembly, align and produce a phylogeny of all retrieved sequences, and detail the mutations with respect the query sequence. A list of the flags available in Hound, and their functionality, can be found in Table 1.

**Table 1:** Options available in Hound, as shown by the command HoundAnalyser–help

Flag	Description
-h, —help	Show this help message and exit
—preprocess FILE DIRNAME	
	Unzip Illumina reads and create appropriate directory structure. It requires a name to create destination directory. REQUIRED unless —project is given.
—project DIR	Directory where FASTQ files can be found. It can be a directory of directories if FASTQ files are contained in a ‘reads’ directory. Maximum directory depth is 2. REQUIRED unless —preprocess is given.
—assemble	Assemble reads. Requires —project.
—reference FILE	Reference genome in FASTA format. Requires —assemble.
—de-novo	Assemble reads de novo. Used to assemble genomes and specify assembly type for data analysis. Requires —assemble.
—coverage	Compute coverage depth to estimate gene copy number. Can be used to assemble genomes or include coverage depth in the data analysis. Requires —hk-genes.
—hk-genes FILE	List of Multilocus sequence typing (MLST) genes, or other reference genes, in FASTA format to compute baseline coverage depth. Requires —coverage.
—genes FILE	List of genes to be found, in FASTA format. Requires —identity and —prefix.
—genes-dir DIR	Directory containing genes of interest in FASTA format. Requires —summary.
—nucl	Use nucleotide sequences for the search. Requires —genes.
—prefix NAME	Label added to all output files. Required to do multiple searches with the same assemblies.
—identity NUM NUM	Identity threshold required to shortlist sequences found. Two floats (min identity, max identity) between 0 and 1 are required. Requires—genes.
—promoter	Isolate the promoter region of the target gene(s) sequences found and ignore coding sequences. Requires —cutoff.
—cutoff NUM	Length of the promoter in nucleotides. Requires —promoter.
—phylo	Align sequences of promoter/coding sequences found, and generate the corresponding phylogeny.
—phylo-thres NUM	Remove sequences that are a fraction of the total size of alignment. Used to improve quality alignment. Requires a number between 0 and 1 (defaults to 0.5).
—plot FILE	Generate plot from the multiple alignment of sequences found, and save as FILE.
—roi FILE	Sequences of interest to look for in the gene(s) found, in FASTA format. Requires—plot.
—summary FILE	Save Hound analysis as a spreadsheet. Requires—project.
—labels FILE	XLS file containing assembly name (col 1), and assembly type (col 6) to label phylogeny leafs (defaults to assembly name). Requires —plot.
—force	Force re-generation of phylogeny and/or plot even if they already exist.

The main drawback of *de novo* assemblies [19] is the fragmentation of genes, with subsets of their sequence being split across two or more contigs by the assembler. In this case, if the identity of the sequences found by Hound are *at least* 90% ($MIN_ID $\ge$ 0.9) to the user-given query, and the contigs harboring subsets of the query have overlapping common sequences, Hound will shortlist the contigs for its contig-merging routine. Here, after sorting them first by identity and length, Hound will iteratively run pairwise alignments between the first pair with overlapping coordinates, discard one of the two overlapping sequences to avoid introducing duplications, and compare the resulting sequence to the user-given query. Hound will process the next contig in the list and add it to the previous merged sequence until the reconstructed sequence has, at least, equal length to the query which will have at least $MIN_ID identity. Note all these contigs will have overlapping coordinates that are located at the boundaries of the contigs, thus, only genes truncated by the assembler and not by insertion sequences—mobile elements—will be included in this analysis.

At this point, once the query sequence can be reconstructed, the assembly is re-written with the new contig name being a concatenation of all the founding contigs (i.e. >NODES_1 + 43 + 24). If coverage data exists, the coordinates used earlier by the merging routine are applied to this dataset to preserve coverage in the new, merged contig.

### Reference-mapped versus *de novo* assemblies

**Figure 2:**
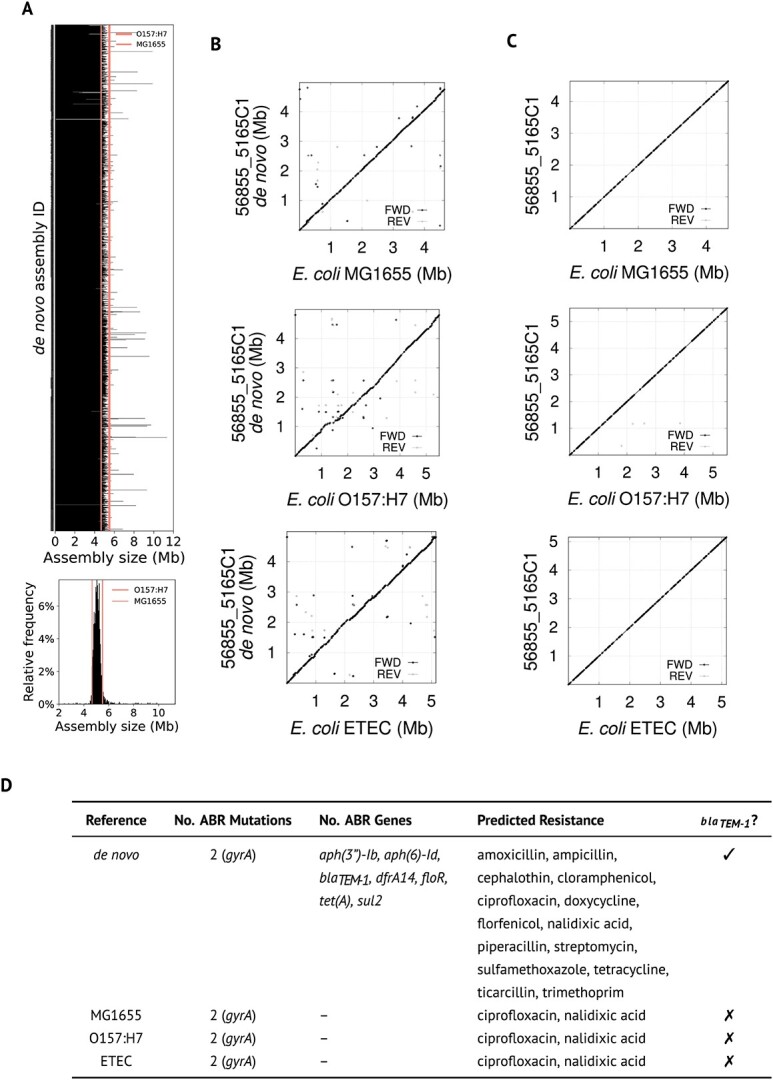
Variability of within species genome size is not captured by the canonical use of reference genomes. (**A**) Histogram (top) and distribution (bottom) of genome sizes across 3562 farm and clinical *E. coli* isolates. The canonical size of the reference wild-type (K-12 substr. MG1655, genome GCF_000005845.2 in the NCBI) and Shiga toxin-producing strain (0157:H7 str. Sakai, genome GCA_000008865.2 in the NCBI) are marked at ~4.64 Mb and 5.59 Mb by vertical lines. (**B**) and (**C**) Dotplots of one representative isolate to visualize the genome–genome sequence alignment between de novo (B) or reference-mapped (C) assemblies and three different known genomes: MG1655, O157:H7, and enterotoxigenic *E. coli* (ETEC). Sequences present in these references, but not in out isolate, are noted by a horizontal gap in the dotplot, whereas the converse is noted by a vertical gap. Note that in C) the reported assembly size of our isolate, in the y-axis, varies depending on the choice of reference—size is constant in those genomes assembled de novo. (**D**) Exemplar report from ResFinder when used against the isolate 56855_5165C1 from B to C assembled de novo, and mapped to different references. This tool reports documented ABR mutations and genes, as well as predicting resistance to certain antibiotics as reported by the literature. Note that, for the de novo assembly, ResFinder reports resistance to ampicillin and amoxicillin. The number of copies of *bla_TEM-1_* reported by Hound is 2–3, thus, the isolate would also be resistant to amoxicillin/clavulanate [28] which is missed by ResFinder. The full report can be found in the supplementary data.

The first step in screening recently acquired genomes of our 5032 bacterial isolates from farmed animals and human clinical samples, purified prior to sequencing, is the assembly *de novo* of the reads. A first look at the output of the pipeline reveals the assemblies seldom have the same size (Figure 2A). Now, while *E. coli* is by far the most abundant species in our dataset, as identified by Kraken 2 [36], the dataset also contains isolates of *Klebsiella pneumoniae* (15.7% of the total), and one isolate of *Pseudomonas aeruginosa* ($\ll$1%) and *Salmonella enterica* ($\ll$1%) among the human clinical isolates. This means multiple species can be monitored depending on each use case.

When we removed all non-*E. coli* from the dataset and compared the assembly sizes, we found the variation with respect to the available reference genomes—K-12 MG1655 and 0157:h7 Sakai—to be substantial as the histogram in Figure 2A shows, with most assemblies having sizes in-between these references. These two genomes are the only ones validated by the National Center for Biotechnology Information (NCBI) and flagged as reference genomes accordingly. The variability that we observed in genome sizes is consistent with the aforementioned notion of pan-genomes. Hound supports the assembly of reads based on a reference genome. So, next, we compared reference-mapped assemblies using to different *E. coli* genomes using Hound with flags—assemble—reference $REF_GENOME with their respective reference—beyond the above MG1655 and 0157:h7 genomes we also used that for enterotoxigenic *E. coli* (ETEC)—as well as the *de novo* assemblies, using pairwise alignments with MUMmer [37]. Note that reference-based assemblies rely on SAMtools and the Burrows-Wheeler aligner, which are implemented in Hound, sharing the downstream analysis pipeline.

When comparing the *de novo* assemblies to the references, this alignment revealed signatures of small deletions, insertions and repeated regions that were different with each reference used (Figure 2B). However, the signatures vanished when we used reference-mapped assemblies (Figure 2C). This means the use of references discards or includes details from the final assembly that can ultimately alter its size—unique and robust when assembled *de novo*—and help explain the inconsistent results [38] that occur when comparing different tools.

### Monitoring *bla*_*TEM-1*_ in farm and clinical isolates

**Figure 3:**
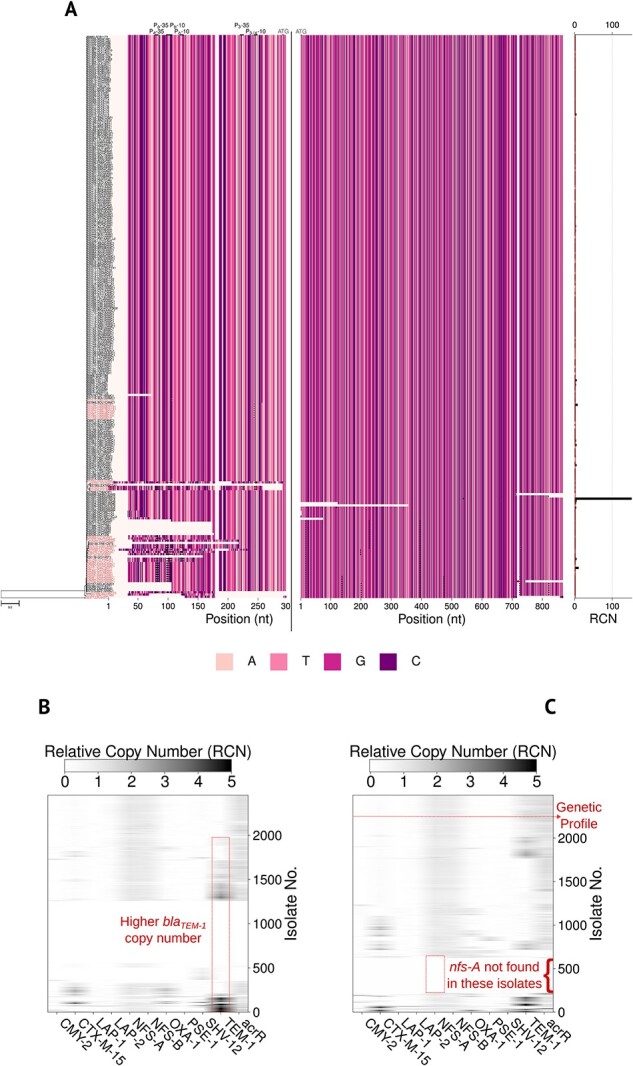
Screening *bla_TEM-1_* across thousands of bacterial isolates. (**A**) Exemplar of a summary plot generated by Hound to show phylogeny (left), multiple alignment of the promoter and coding sequence (center), and relative copy number (RCN, right) of a subset of the farmed animals data positive for *bla_TEM-1_*. This is to help visualize details of the plot. A dot is placed on positions where the nucleotide sequence deviates from the consensus sequence (present in at least 80% of the isolates). Note these are mutations within the multiple alignment, the mutations reported in Hound’s spreadsheet are those from pairwise alignments between the sequence in each isolate and the user-given target sequence and the resulting change in amino acid. Isolates with mutations are highlighted in red by default. When regions of interest have been given alongside the promoter flag, they are added at the top of the alignment (here are promoter regions P_a_-35, P_b_-10, P_3_–35, P_3/4_–10), and start codon. (**B**) and (**C**) Heatmap created *ad hoc* to summarize our search of *bla_TEM-1_* in all 5032 isolates with Hound. The presence or absence is noted by a shaded horizontal line in the heatmap, and the relative copy number is noted by different shade intensities—with darker denoting isolates with more copies and lighter those with fewer copies. Note the absence of genes such as *nfs-A* and *nfs-B* exposes the of that are not *E. coli*. Similarly, heatmaps reveal that genes like *lap-1* or *lap-2* are more frequent in farm isolates. In general, the horizontal of these heatmaps represent the genetic profile of a given isolate—here to infer resistance to the combination of amoxicillin/clavulanate.

We used this tool to screen through our 5032 isolate whole-genome sequencing datasets, from farmed animals (2494) and human clinical samples (2538), to detect *bla*_TEM-1_ (including non-synonymous variants), its promoter region, and relative copy number (Figure 3). Among the output files generated by Hound, there is a figure with the multiple alignment and phylogeny of the sequences, containing the aforementioned metadata depending upon the flags provided, that can be produced with the flag—plot $FILENAME. This is an exemplar of how this tool can improve the prediction and, therefore, the surveillance of antibiotic resistance with complex phenotypes that are notoriously difficult to predict from mutations in the coding sequence alone.

The result shows 39.16% (*n* = 994) of clinical isolates were *bla_TEM-1_* positive compared to 51.84% (*n* = 1283) in those from farm animals (Figure 3B and C). It is noteworthy to mention that human clinical isolates largely came from routine surveillance of Gram-negative bacteria and include multiple species beyond *E. coli* that less commonly carry *bla_TEM-1_*, and would also include isolates resistant to very few antibiotics, whereas those from farmed animals were *E. coli* isolates sequenced due to their resistant phenotype as recently reported [39]. Interestingly however, only 22.52% (*n* = 289) of the isolates from farmed animals harbored two or more copies of the gene. This contrast with data from the clinical isolates, where 39.33% of the *bla_TEM-1_* positive isolates (*n* = 391) harbored two or more copies. Since increased gene copy number is associated with amoxicillin/clavulanate resistance [31], this would fit with a higher rate of resistance to this combination given its more widespread use in the clinic to treat humans. Moreover, as Figure 3A and B illustrate, in some isolates *bla_TEM-1_* copy number is in the hundreds. While their occurrence is rare—*n* = 6 in farm isolates, *n* = 31 in clinical isolates—it suggests the circulation of one or more *bla_TEM-1_*-encoding plasmids with very high copy number. Indeed, multicopy plasmids with hundreds of copies are not unheard of [40]. Using the flag—promoter—cutoff 250 we used Hound to retrieve the promoter of *bla_TEM-1_* and flag any mutation found. Again, mutations associated with over-expression, annotated in the figure produced as black dots, were more common in isolates from humans than farmed animals as Figure 2B and C illustrate. Mutations found by Hound with respect to the query sequence used, as well as relative copy number and other metadata can be exported into a spreadsheet by using the flag—summary $SPREADSHEET, which can then be parsed to highlight isolates with specific mutations. An illustrative spreadsheet is included as a Supplementary Table 1.

A useful feature of Hound is its ability to populate iteratively the same assemblies to look for different genes, allowing the simultaneous detection of genes, calculation of their relative copy number, or re-analysis of prior data. Figure 2D shows two heatmaps illustrating the detection of different $\beta -$lactamases as well as nitroreductases, and efflux pumps—where mutations affecting their production can cause resistance to multiple antibiotics [41]—in both farm and clinical isolates as well as any mutations found in their coding sequence and relative copy number.

## DISCUSSION

An increasingly problematic issue, particularly in the detection of ABR genes, is the lack of reproducibility [42, 43]. The choice of reference genomes is typically opaque to the user beyond the species, being notoriously difficult to underpin the exact assembly used. Along with the variety of existing pipelines that yield inconsistent results between laboratories [38], this problem led to the suggestion of standardized, ISO-certified pipelines [38]. Here we argue that they still fail to detect antibiotic resistance driven by the over-expression of enzymes like $\beta -$lactamases given their inability to account for gene over-expression caused by promoter mutations and, particularly, increase in gene copy number. Now, beyond the detection of antibiotic resistance mutants, if we found different genetic signatures when comparing our assemblies to different references it is not unreasonable to think this will also be the case for other reference-mapped assemblies. Thus, using a standardized reference is unlikely to avoid this problem—exacerbated by the scarcity of tools to analyze *de novo* assemblies.

Hound is a step toward facilitating the analysis of these assemblies, not only by addressing a key limitation—gene fragmentation—but also by reducing the knowledge and technological burdens. The fact that we assembled and analyzed ˃5000 genomes on an 8-core, 16GB RAM laptop over the span of 9–10 days shows the potential for Hound to be implemented in larger and more powerful infrastructures for surveillance and diagnostic purposes. Now, Hound has some limitations. For example, it cannot report whether a gene of interest sits within a genomic island, but it can be used to detect whether genes associated to such islands are in the same contig as the gene of interest—complementing other bespoke analyses. Another limitation is that it currently only supports short-read Illumina sequencing data due to its availability during the development of this tool. However, given our use of SPAdes as backend assembler it is possible to add support for long-read nanopore and PacBio sequencing data in future releases. A similar argument can be for metagenomic datasets. While here all isolates were purified, SPAdes supports metagenomic data, only requiring the corresponding implementation in Hound. Given our limited access to such datasets, we could not test this implementation.

The use of *de novo* assemblies means Hound is not only agnostic with respect to which genes can be monitored. It is also independent of the microbial species analyzed—not possible with the use of reference genomes. With the majority of prokaryote diversity still being unknown and unsequenced [44, 45], we believe that Hound can be a useful tool to study non-model microorganisms that lack any reference—and help build them iteratively thanks to its contig-merging routine when sequencing costs in other platforms increase.

## METHODS

### Bacterial isolation

Prior to screening *bla*, we grew all samples (clinical or from farm sources) in chromogenic agar (Chromagar, Paris) at 37°C for 24 h, using Tryptone Bile X-Glucunoride (TBX) to aid purification and identification of *E. coli*. We confirmed speciation of clinical isolates with MALDI-TOF mass spectrometry (Bruker Microflex). Prior to sequencing, we grew *E. coli* in TBX agar and all other species in Nutrient Agar (Oxoid).

### Genome sequencing and assembly

Genomic DNA libraries from isolate bacteria were prepared using the Nextera XT Library Prep Kit (Illumina, San Diego, USA) following the manufacturer’s protocol with the following modifications: The input DNA was increased 2-fold with respect to the manufacturer’s protocol, and the Polymerase Chain Reaction (PCR) elongation time was increased to 45 s. DNA quantification and library preparation were carried out on a Hamilton Microlab STAR automated liquid handling system (Hamilton Bonaduz AG, Switzerland), and the libraries sequenced on a Illumina NovaSeq 6000 (Illumina, San Diego, USA) using a 250 paired-end protocol by MicrobesNG. Reads were adapter-trimmed using Trimmomatic 0.30 [46] with a sliding window quality cutoff of Q15.

The flag—preprocess reads.zip processes the reads provider by MicrobesNG to create the directory structure required by Hound, with paired reads being stored in $DIR/reads/, and assemblies in $DIR/assemblies/de_novo/ for reads assembled *de novo* or $DIR/assemblies/reference-mapped/ for those mapped to a reference. This will depend on whether—assembly—de-novo or—assembly—reference $REF_GENOME have been passed. Hound then assembles *de novo* the resulting reads using SPAdes with the—isolate flag and $k$-mer size of 127, given the sequencing platform and protocol. For reference-mapped assemblies, Hound aligns the reads to a user-given reference using the Burrows-Wheeler Aligner and SAMtools with standard parameters. The coverage depth for all assemblies is then calculated with SAMtools if the flags—coverage and—hk-genes $HK_GENES are given, the baseline depth being the median coverage depth of all loci included in $HK_GENES, faster than computing the median coverage of the whole assembly to avoid any bias introduced by plasmid carriage. The relative gene copy number (RCN) is then calculated as the coverage depth of all loci in $TARGET_GENES divided by the baseline coverage [47].

### Indexing of assemblies

The resulting assemblies are indexed using makeblastdb from BLAST+, with flags -parse_seqids and -dbtype nucl, to facilitate the search of the sequences in file $TARGET_GENES. The search is run with blastn, which uses nucleotide sequences, or tblastn depending on whether the flag —nucl is passed to Hound. Without this flag, Hound assumes that the file $TARGET_GENES contains amino acid sequences and will therefore use tblastn.

### Multiple alignment and phylogeny

When the flag—phylo is passed, Hound will use muscle 3.80 to align the target gene sequences found in all assemblies given its accuracy and speed [48]. Penalties for the introduction and extension of gaps are pre-set with a value of −9950.0 to avoid excessive fragmentation of the alignment. This alignment is then used by Hound to generate a phylogeny with PhyML [49] with seed 100 100 for repeatability.

### Implementation details

Hound was developed on a laptop with an x86–64 processor (AMD Ryzen 6900HS, 8-cores/16-threads) and 16GB of DDR5 Random Access Memory (RAM), running ArchLinux and using GNOME Builder. The software is written in Python3, requiring at least Python v3.9 and the libraries numpy [50], matplotlib [51], biopython [52]. For plotting the phylogenies, we used ete3 [53] through its Python API. We installed and tested Hound on Intel-(4-cores) and M1-powered (8-cores) Apple Macbook Pro to confirm compatibility with macOS. The code available through the gitlab repository below produces a Wheel file (.whl) that will automatically retrieve all Python dependencies upon installation with Python’s pip package manager. External software such as SAMtools, Burrows-Wheeler aligner, SPAdes, the BLAST suite and muscle, must be provided independently and added to the system $PATH.

**Key Points**

- Standard tools and databases associate genotypes with the presence/absence of specific genes, or mutations in their coding sequence, failing to detect those caused by the amplification or mutations that lead to over-expression of such genes. Hound can help detect such mutants by reporting promoter mutations, along with the relative abundance of the user-given sequence within an assembly. Importantly, Hound requires amino acid sequences to only report those mutations, in the coding sequence, that could lead to a change in function.
- Reference-guided assemblies mask the rich genetic diversity of bacteria, and we showed that the final assembly can change only by virtue of the reference chosen
- The main drawback of *de novo* assemblies is the fragmentation of genens during the assembly process. To circumvent this problem, Hound uses use the user-given query as a template to aid its contig-merging routine. This occurs when the identity between user-given query and hit within the assembly have an identity of 90% or higher. The result is fewer, but larger contigs where the amino acid sequence entered by the user can be unequivocally reconstructed.
- Hound produces a spreadsheet summary that can be screened to automatically highlight mutations of interest, depending on the genotype sought after—here being the increase copy number of *bla_TEM-1_* and specific promoter mutations.

## Supplementary Material

ResFinder_bbae057

Sample_Summary_bbae057

## Data Availability

Whole-genome sequencing data have been deposited in the European Nucleotide Archive under project accession numbers PRJEB45949, PRJEB70722 and PRJEB72122.

## CODE AVAILABILITY STATEMENT

A Python implementation of Hound, licensed under the GNU Lesser General Public License (LGPL) 2.1, can be found in https://gitlab.com/rc-reding/software/

## Author Biographies

**Carlos Reding** is a senior research associate at the University of Bristol. His research focuses on microbial evolution and development of quantitative methods.

**Naphat Satapoomin** is a PhD candidate at the University of Bristol supervised by Prof. Matthew Avison. Her research focuses on the characterization of resistance to $\beta$-lactam/$\beta$-lactamase inhibitor combinations.

**Matthew B. Avison** is a professor at the University of Bristol. His research aims to characterize multi-drug resistance mechanisms in Gram-negative bacteria.
